# Supplementary material for: The voltage-gated potassium channel Shaker promotes sleep via thermosensitive GABA transmission
Source: Commun Biol. 2020 Apr 15;3:174. doi: 10.1038/s42003-020-0902-8 (PMC7160125; doi:10.1038/s42003-020-0902-8)
Supplement: Supplementary file 1 — Supplementary Information [file 42003_2020_902_MOESM1_ESM.pdf]

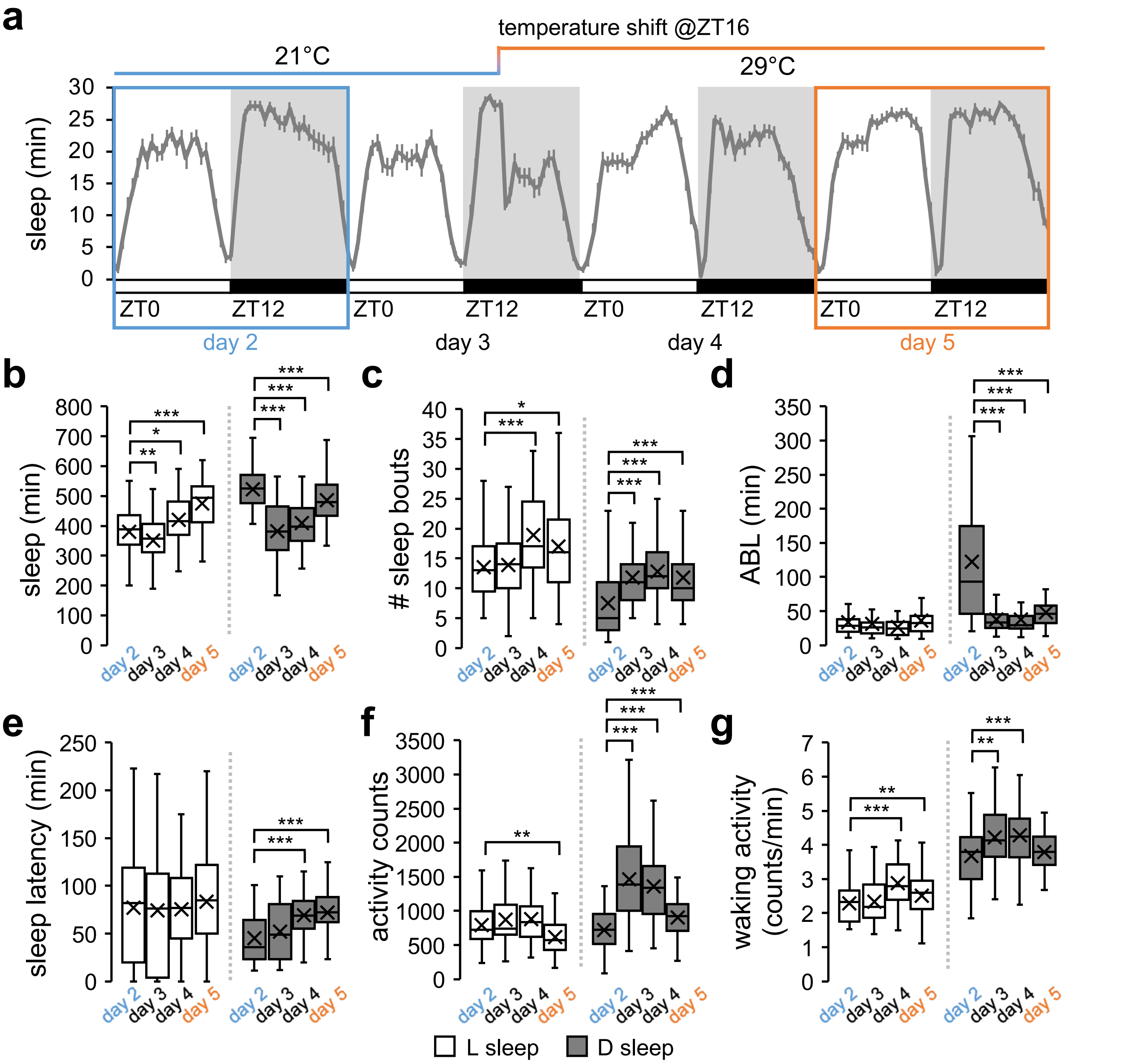

Supplementary Fig. 1 Temperature-sensitive plasticity of *Drosophila* sleep. **a** Sleep behaviors in wild-type male flies were individually monitored in 12-hour light (white bars): 12-hour dark (black bars) cycles. Ambient temperature was shifted from 21°C to 29°C as depicted at top. Data represent average +/- SEM (n=57). ZT, zeitgeber time (lights-on at ZT0; lights-off at ZT12). **b-g** Daily sleep amount, total sleep bout numbers, ABL (averaged sleep bout length), sleep latency (total minutes awake from lights-on or lights-off to the first sleep bout), total activity counts, and waking activity (activity counts per minute awake) were analyzed in individual flies and averaged per day (n=57). Each box plot ranges from lower Q1 to upper Q3 quartile; crosses and horizontal lines inside each box indicate mean and median values, respectively; whiskers extend to minimum or maximum values of 1.5 x interquartile range. White boxes, L sleep parameters; gray boxes, D sleep parameters. **P* < 0.05, ***P* < 0.01, ****P* < 0.001 as determined by Aligned ranks transformation ANOVA, Wilcoxon signed rank test.

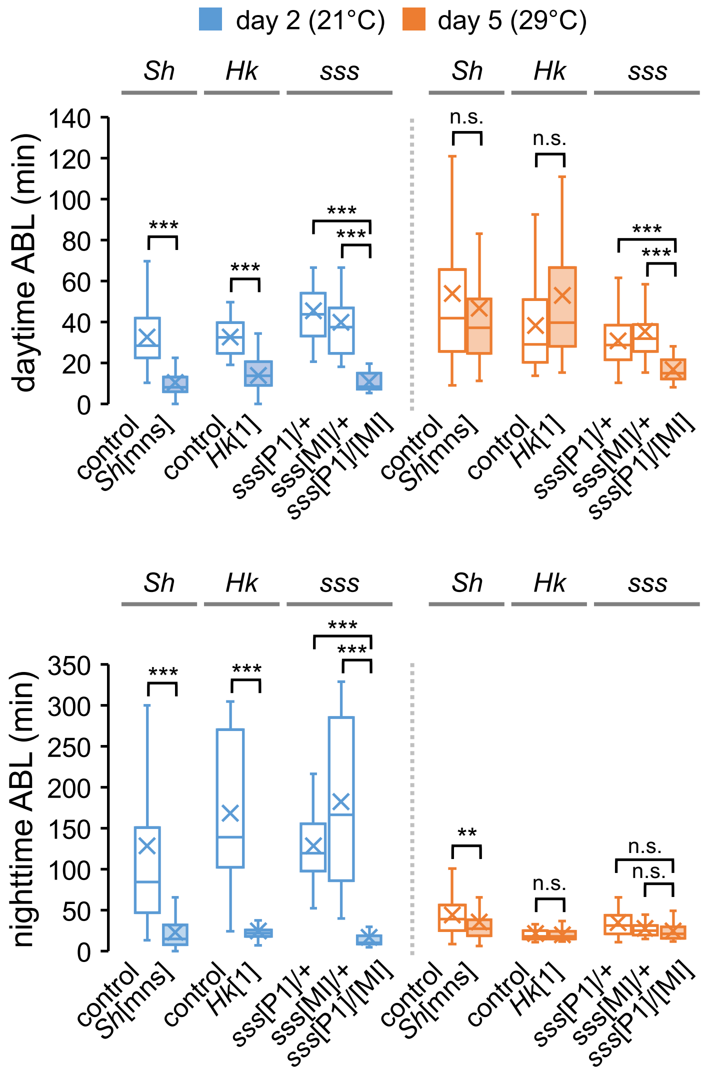

Supplementary Fig. 2 High temperature rescues L sleep fragmentation in *Sh* and *Hk* mutants. Sleep behaviors were analyzed in individual male flies as described in Fig. 1. Box plots represent daytime or nighttime ABL on day 2 (21°C, blue boxes) versus day 5 (29°C, orange boxes) (n=20-122). Aligned ranks transformation ANOVA detected significant interactions of temperature with *Sh*[mns], *Hk*[1], or *sss*[P1]/*sss*[MIC] on daytime ABL ($P < 0.0001$ for *Sh*; $P < 0.001$ for *Hk*; $P < 0.05$ for *sss*) and on nighttime ABL ($P < 0.0001$ for all genotypes). n.s., not significant; ***P* < 0.01, ****P* < 0.001 as determined by Wilcoxon signed rank test.

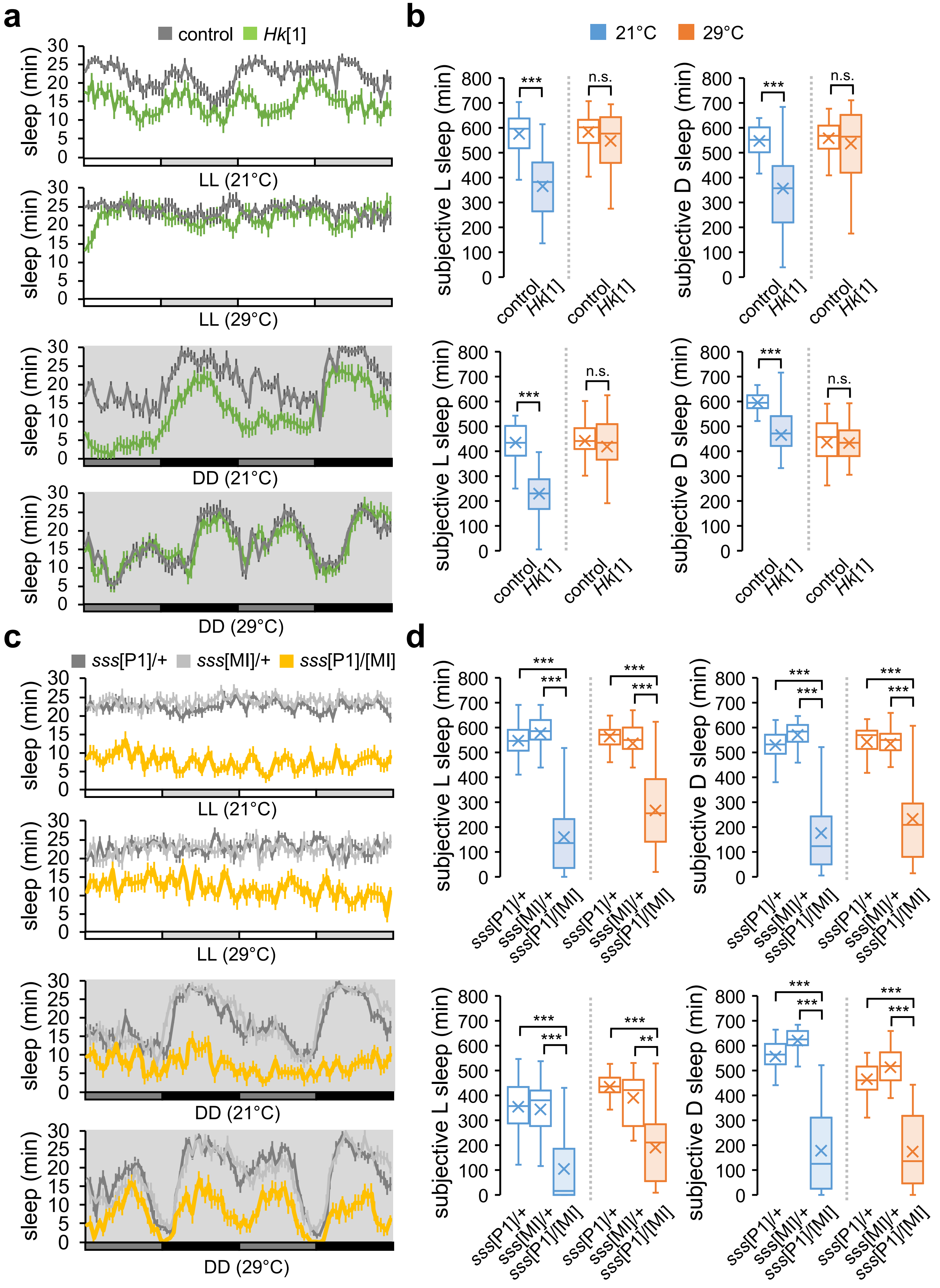

a
control Hk[1]
sleep (min)
LL (21°C)
LL (29°C)
DD (21°C)
DD (29°C)
b
21°C 29°C
subjective L sleep (min)
subjective D sleep (min)
control Hk[1]
***
n.s.
c
sss[P1]/+ sss[MI]/+ sss[P1]/[MI]
d
sss[P1]/+ sss[MI]/+ sss[P1]/[MI]
**

Supplementary Fig. 3 *Hk* and *sss* mutants exhibit differential temperature sensitivity of their sleep behaviors in constant conditions. Sleep behaviors in individual male flies were analyzed in constant light (LL) or constant dark (DD) at 21°C or 29°C. **a**, **c** Sleep profiles of control (gray lines), *Hk* mutants (green lines), or *sss* mutants (yellow lines) during the first two cycles of LL or DD. Data represent average +/- SEM (n=24-47). **b**, **d** Box plots represent the total amounts of subjective L or subjective D sleep on the second cycle of LL or DD at 21°C (blue boxes) or 29°C (orange boxes). Aligned ranks transformation ANOVA detected significant interactions of temperature with *Hk* mutation on sleep duration in either LL or DD ($P < 0.0001$ for all conditions), but not with *sss* mutation ($P > 0.05$ for all conditions). n.s., not significant; **$P < 0.01$, ***$P < 0.001$ as determined by Wilcoxon rank sum test.

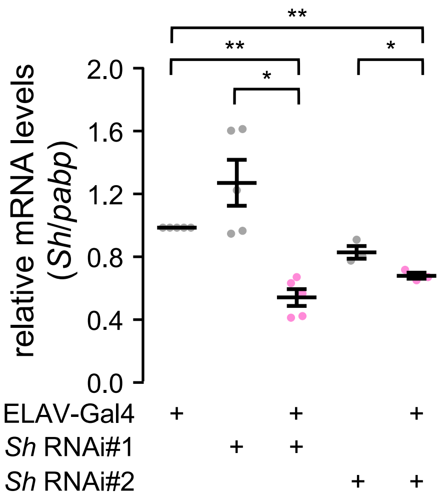

Supplementary Fig. 4 Pan-neuronal over-expression of the *Sh* RNA interference (RNAi) transgenes depletes endogenous *Sh* mRNAs in fly head extracts. Total RNAs were prepared from male fly heads. Abundance of each transcript was quantified by real-time RT-PCR and normalized to that of *polyA-binding protein* (*pabp*). Relative mRNA levels were then calculated by normalizing the relative abundance of *Sh* mRNAs in each genotype to that in ELAV-Gal4 control (set as 1). Two independent transgenes for *Sh* RNAi (*Sh* RNAi#1, BL53347; *Sh* RNAi #2, v104474) were tested. Data represent average +/- SEM (n=5 independent biological samples for *Sh* RNAi#1; n=3 independent biological samples for *Sh* RNAi #2). *$P < 0.05$, **$P < 0.01$ by one-way ANOVA, Tukey post hoc test.

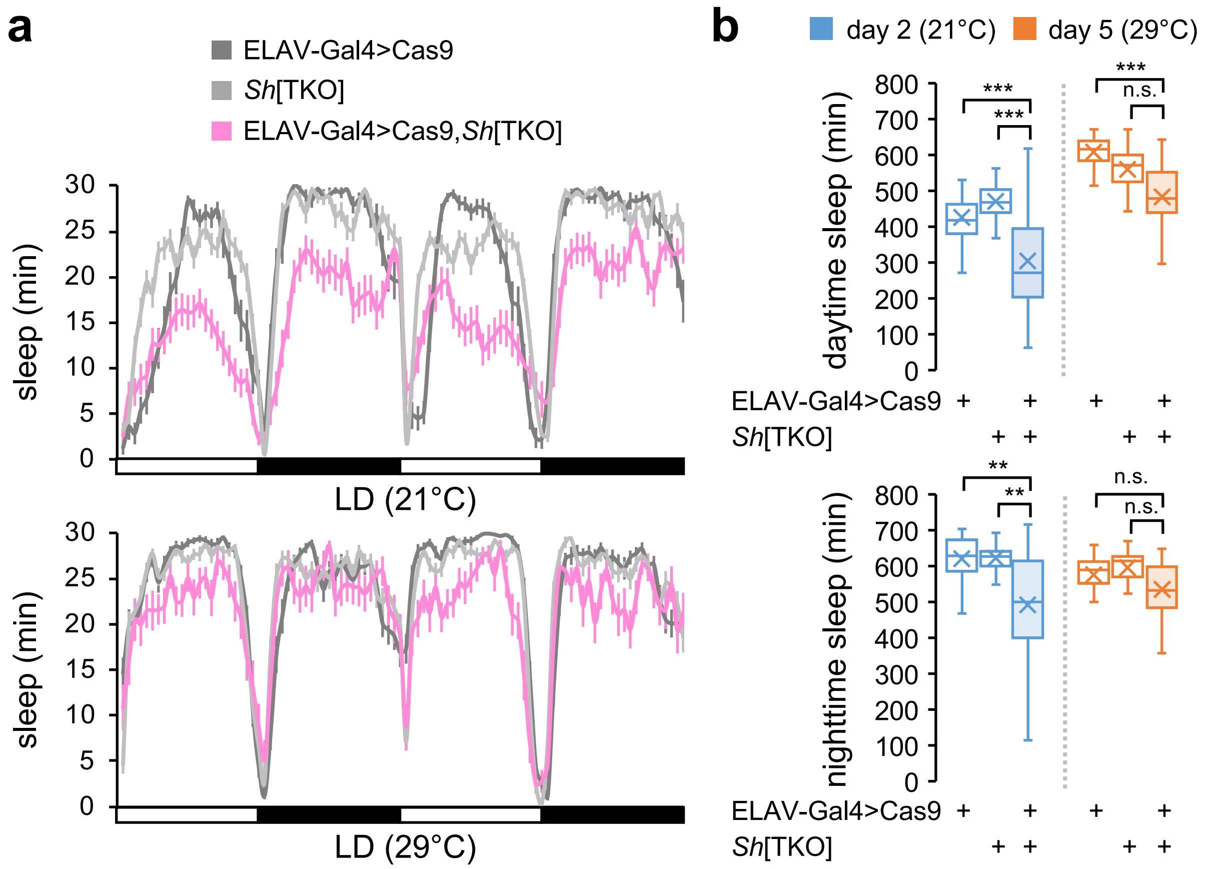

Supplementary Fig. 5 Pan-neuronal disruption of the *Sh* locus by CRISPR-mediated targeting suppresses sleep only at 21°C. A ubiquitously expressed, single-guide RNA transgene that targets *Sh* gene (*Sh*[TKO]) was genetically combined with ELAV-Gal4>Cas9 to induce neuron-specific *Sh* deletion. Sleep behaviors in individual male flies were analyzed in LD cycles at 21°C or 29°C. **a** Sleep profiles of control (gray lines) or pan-neuronal *Sh* deletion mutants (pink lines) during the first two LD cycles. Data represent average +/- SEM (n=18-40). **b** Box plots represent the total amounts of L or D sleep on the second LD cycle at 21°C (blue boxes) or 29°C (orange boxes) (n=18-40). n.s., not significant; ** $P < 0.01$, *** $P < 0.001$ by Aligned ranks transformation ANOVA, Wilcoxon rank sum test.

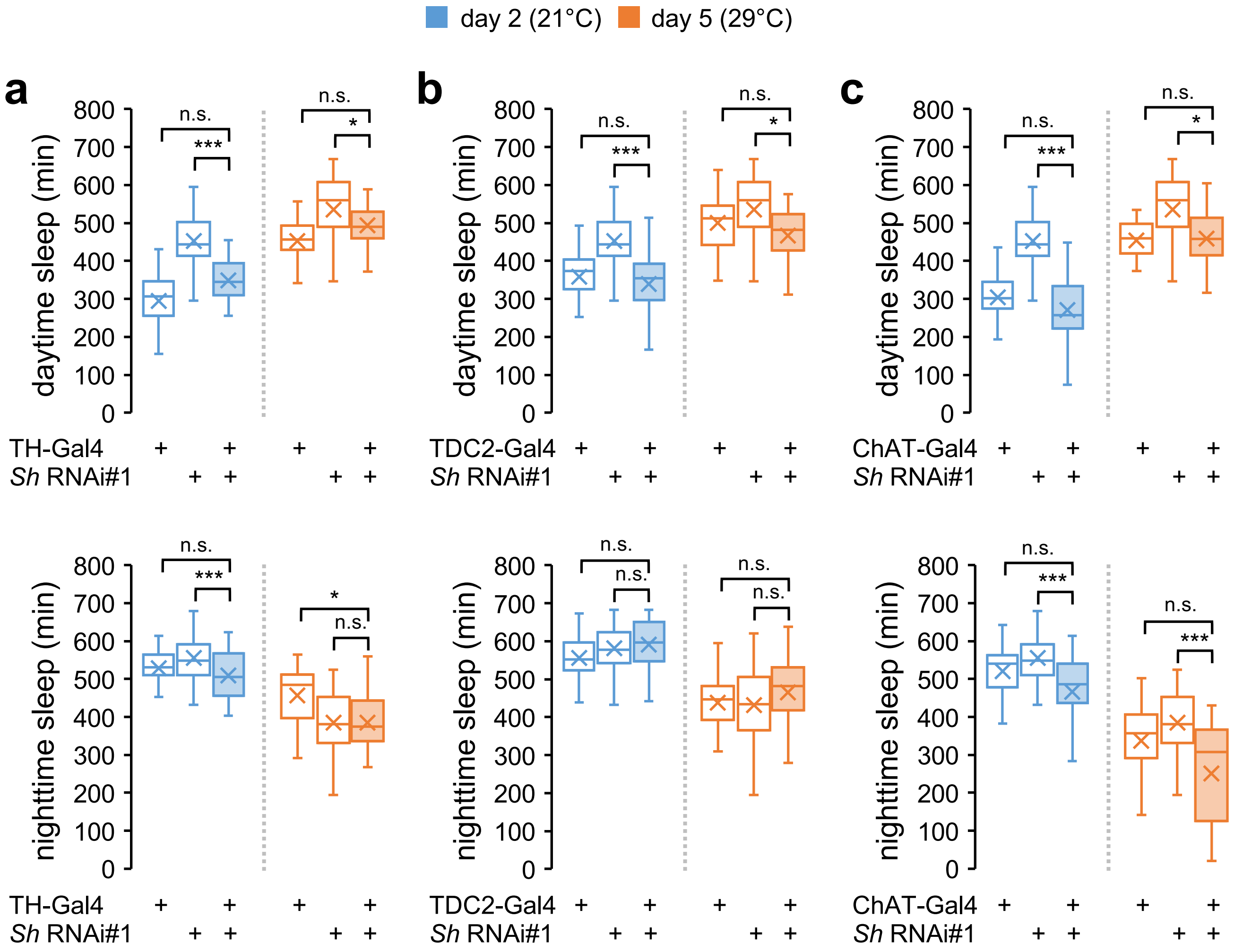

Supplementary Fig. 6 *Sh* depletion in neurons that release wake-promoting neurotransmitters does not significantly affects sleep behaviors. Each Gal4 driver was genetically combined with the *Sh* RNAi transgene to deplete *Sh* expression in dopaminergic (**a**, TH-Gal4), octopaminergic (**b**, TDC2-Gal4), or cholinergic neurons (**c**, ChAT-Gal4). Sleep behaviors were individually analyzed in transgenic male flies as described in Fig. 1. Box plots represent the total amounts of L or D sleep on day 2 (21°C, blue boxes) versus day 5 (29°C, orange boxes) (n=20-62). n.s., not significant; *$P < 0.05$, ***$P < 0.001$ as determined by Aligned ranks transformation ANOVA, Wilcoxon signed rank test.

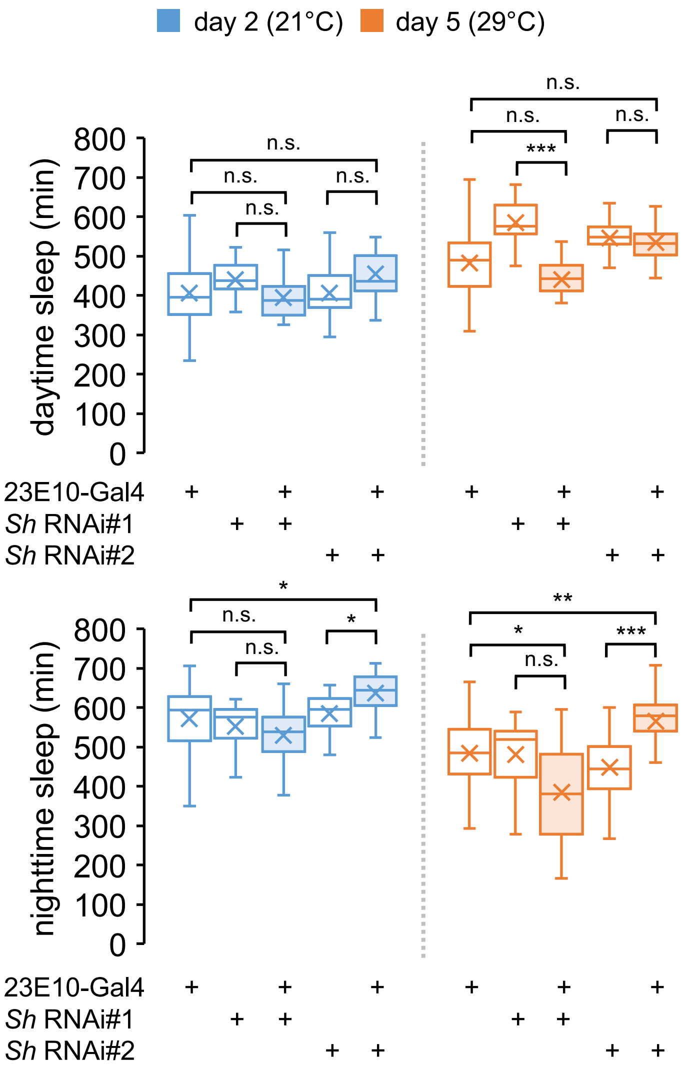

Supplementary Fig. 7 *Sh* depletion in dFSB neurons does not suppress sleep. Each *Sh* RNAi transgene (*Sh* RNAi#1, BL53347; *Sh* RNAi #2, v104474) was overexpressed by 23E10-Gal4 to deplete *Sh* expression in dFSB regions. Sleep behaviors were individually analyzed in transgenic male flies as described in Fig. 1. Box plots represent the total amounts of L or D sleep on day 2 (21°C, blue boxes) versus day 5 (29°C, orange boxes) (n=20-52). Similar results were obtained when the RNAi-enhancing *Dicer-2* was co-expressed (data not shown). n.s., not significant; *$P < 0.05$, **$P < 0.01$, ***$P < 0.001$ as determined by Aligned ranks transformation ANOVA, Wilcoxon signed rank test.

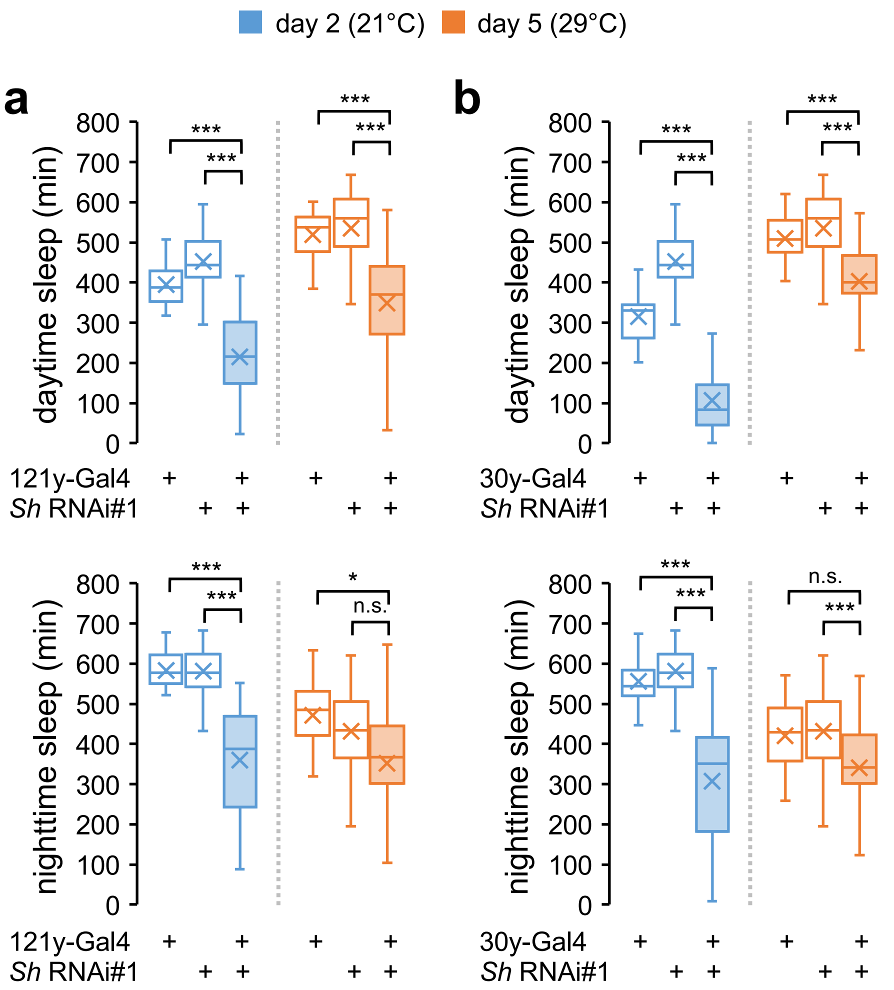

Supplementary Fig. 8 *Sh* depletion in broad subsets of brain neurons causes either temperature-dependent or temperature-independent short sleep. 121-Gal4 (**a**) or 30y-Gal4 (**b**) driver was genetically combined with the *Sh* RNAi transgene to deplete *Sh* expression in broad ranges of brain neurons, including the mushroom body. Sleep behaviors were individually analyzed in transgenic male flies as described in Fig. 1. Box plots represent the total amounts of L or D sleep on day 2 (21°C, blue boxes) versus day 5 (29°C, orange boxes) (n=23-62). Aligned ranks transformation ANOVA detected significant interactions between *Sh* depletion and temperature on the duration of L sleep ($P < 0.0001$ for 30y-Gal4) or D sleep ($P < 0.05$ for 121y-Gal4; $P < 0.01$ for 30y-Gal4). n.s., not significant; *$P < 0.05$, ***$P < 0.001$ as determined by Wilcoxon signed rank test.

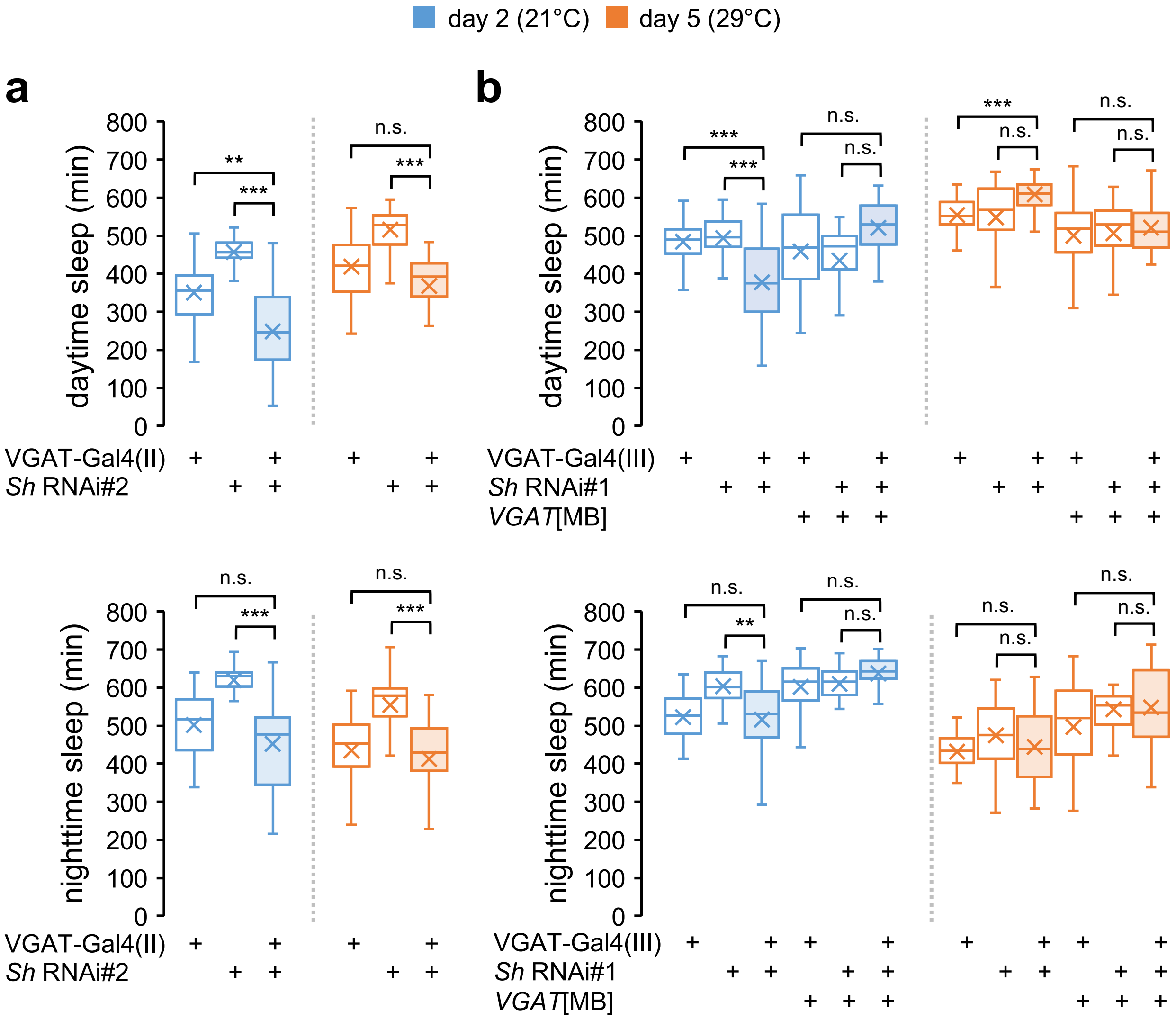

Supplementary Fig. 9 *Sh* acts in GABAergic neurons to promote sleep likely via GABA transmission. **a** An independent *Sh* RNAi transgene (*Sh* RNAi#2, v104474) was co-expressed with the RNAi-enhancing *Dicer-2* to deplete *Sh* expression in GABAergic neurons by VGAT-Gal4 (II, BL58980). Sleep behaviors were individually analyzed in transgenic male flies as described in Fig. 1. Box plots represent the total amounts of L or D sleep on day 2 (21°C, blue boxes) versus day 5 (29°C, orange boxes) (n=23-38). **b** An independent VGAT-Gal4 transgene (III, BL58409) was combined with *Sh* RNAi#1 (BL53347) to deplete GABAergic *Sh* expression in control or *VGAT* heterozygous backgrounds (*VGAT*[MB]/+) (n=23-37). n.s., not significant; ***P* < 0.01, ****P* < 0.001 as determined by Aligned ranks transformation ANOVA, Wilcoxon signed rank test.

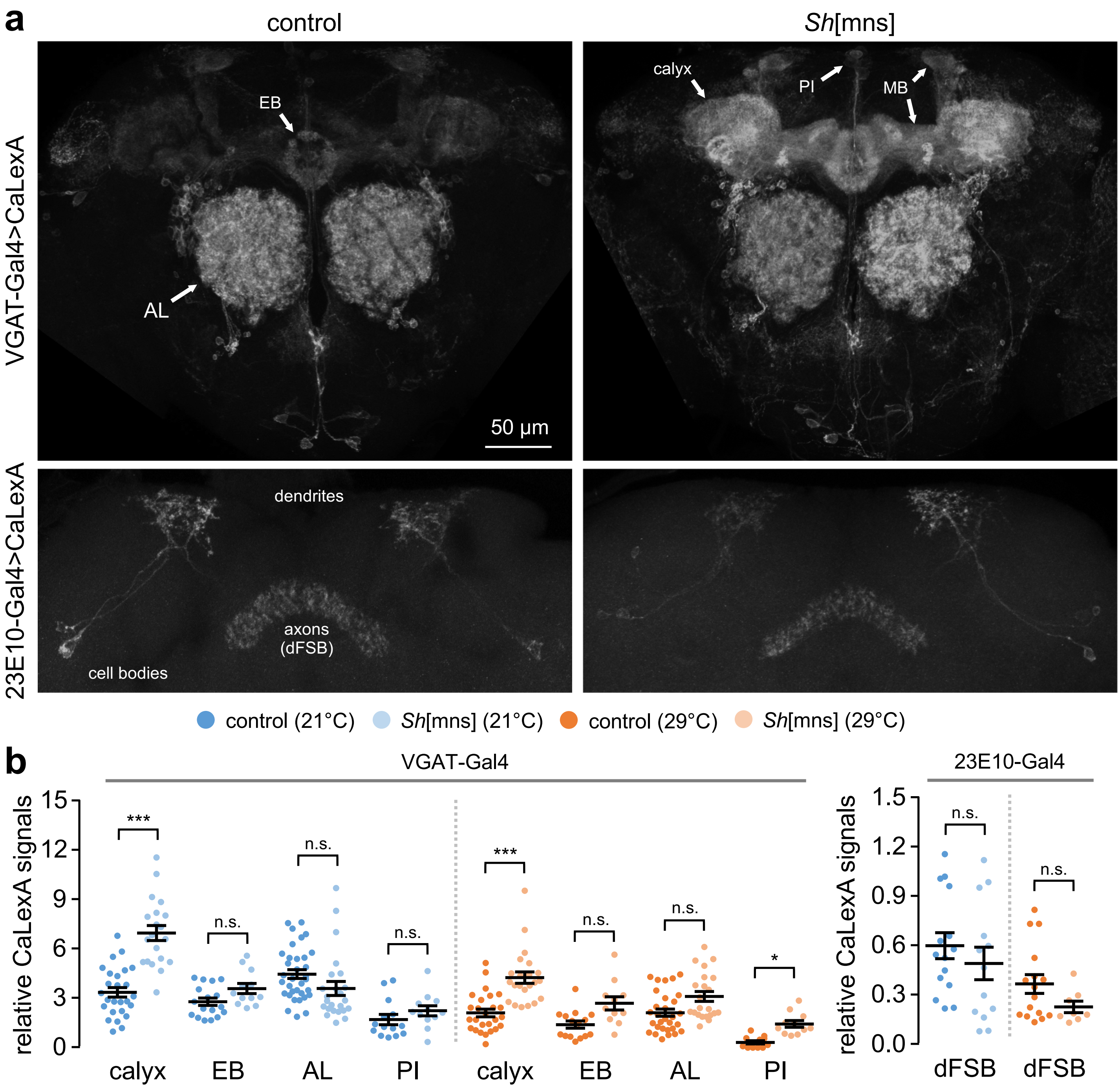

Supplementary Fig. 10 *Sh* mutation elevates intracellular Ca^{2+} levels in selective groups of GABAergic neurons. **a** A transgene encoding a transcriptional fluorescence reporter for intracellular Ca^{2+} levels (CaLexA) was expressed in GABAergic neurons (VGAT-Gal4) or dFSB neurons (23E10-Gal4) of wild-type or *Sh* mutants. After LD entrainment at 21°C or 29°C, adult brains were dissected from transgenic flies and immuno-stained with anti-GFP antibody. Z-stacks of whole-mount brain images were obtained using confocal microscopy. AL, antenna lobe; calyx, a neuropil structure from Kenyon cells that constitute mushroom body (MB); EB, ellipsoid body; MB, axonal lobes projected from Kenyon cells; PI, pars intercerebralis. **b** The fluorescence intensity in individual groups of neurons were quantified using ImageJ software. Relative CaLexA signals were then calculated by the equation (S-B)/B where S and B are the mean fluorescence intensities from each group of neurons and adjacent background regions, respectively. Data represent average +/- SEM (n brains=8-17). n.s., not significant; **P* < 0.05, ****P* < 0.001 as determined by Aligned ranks transformation ANOVA, Wilcoxon rank sum test.

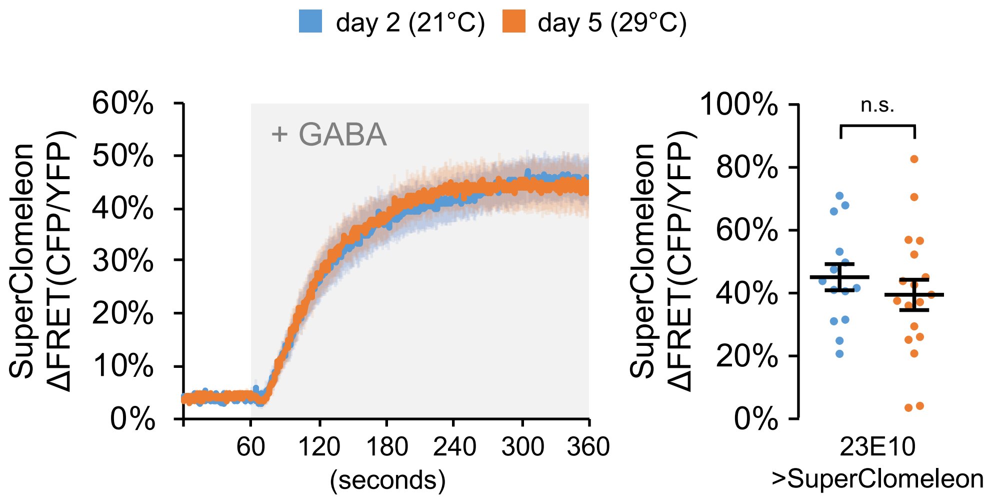

Supplementary Fig. 11 Ionotropic GABA transmission in dFSB neurons is temperature-insensitive. A fluorescence resonance energy transfer (FRET) sensor for Cl^- was expressed in dFSB neurons (23E10>SuperClomeleon) to quantify their GABA-induced chloride influx via ionotropic GABA receptors. Transgenic flies were pre-entrained in LD cycles at 21°C (blue) or 29°C (orange). Whole brains were dissected out, transferred to an imaging chamber, and pre-equilibrated with HL3 buffer for 10 minutes prior to the induction of FRET responses by the batch application of 50 mM GABA (shaded by a gray box). A time series of the fluorescence images was recorded using a multi-photon microscopy and their FRET analysis was performed using ZEN software. Data represent average +/- SEM (n=14 for both temperature). n.s., not significant (P = 0.4004) as determined by unpaired Student's t-test.

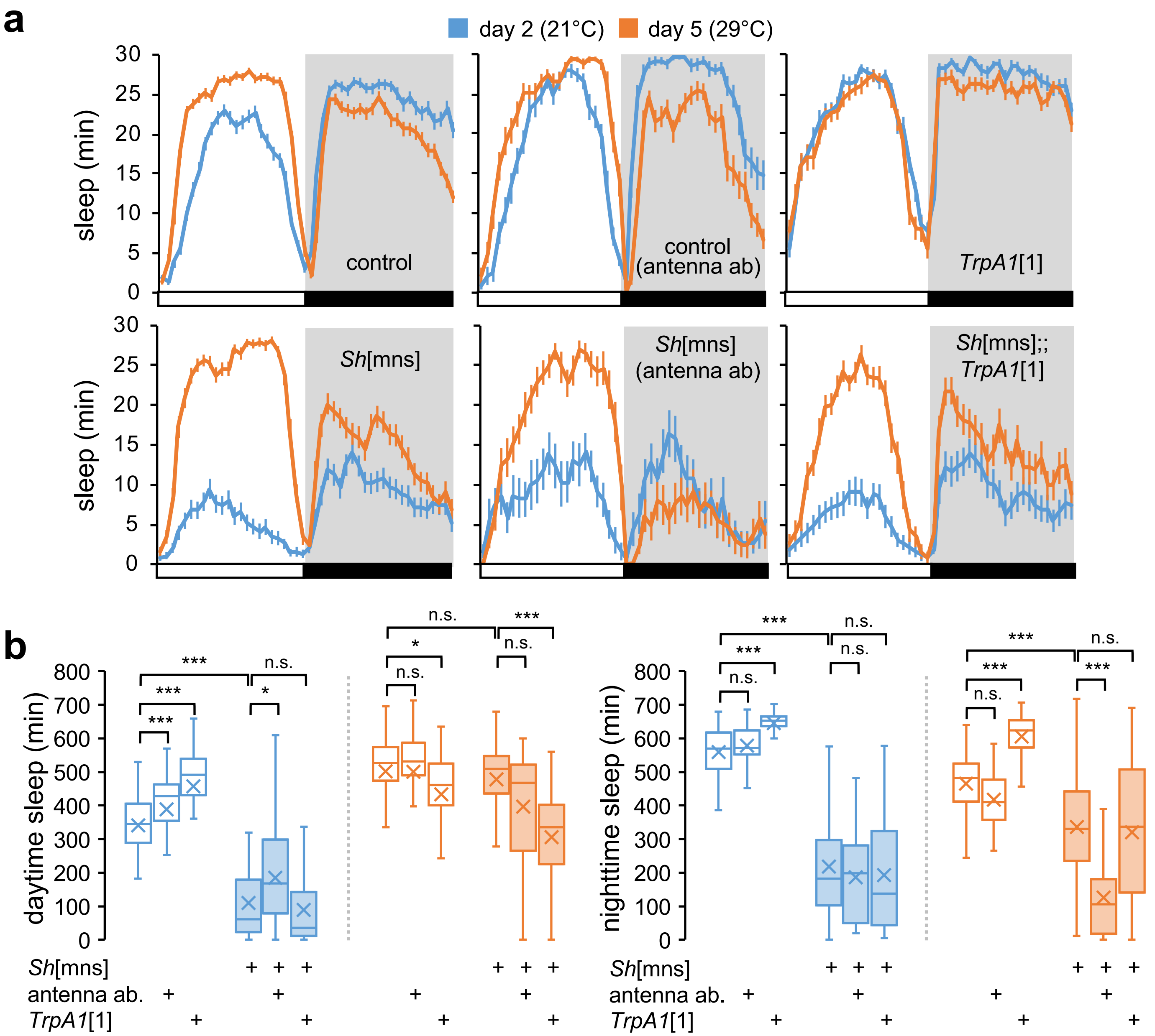

Supplementary Fig. 12 Loss of *TrpA1* function suppresses temperature-sensitive plasticity of wild-type sleep, but not of *Sh* mutant sleep. **a** Sleep profiles of wild-type or *Sh* mutant flies with the surgical ablation of antenna (antenna ab) or with *TrpA1* mutation on day 2 (21°C, blue lines) versus day 5 (29°C, orange lines). Data represent average +/- SEM (n=20-122). **b** Box plots represent the total amounts of L or D sleep on day 2 (21°C, blue boxes) versus day 5 (29°C, orange boxes) (n=20-122). n.s., not significant; **P* < 0.05, ***P* < 0.01, ****P* < 0.001 as determined by Aligned ranks transformation ANOVA, Wilcoxon signed rank test.
