# Supplementary material for: The voltage-gated potassium channel Shaker promotes sleep via thermosensitive GABA transmission
Source: Commun Biol. 2020 Apr 15;3:174. doi: 10.1038/s42003-020-0902-8 (PMC7160125; doi:10.1038/s42003-020-0902-8)
Supplement: Supplementary file 4 — Description of Additional Supplementary Files [file 42003_2020_902_MOESM4_ESM.pdf]

Description of Additional Supplementary Files

File Name: **Supplementary Data 1**
Description: Effects of Gal4-specific Sh depletion on daily sleep amount (Source data for Figure 3a)
